# Editorial: Natural and synthetic microbiology for the production of novel biomolecules for applications in the areas of food, fuel, farming, pharma and environment

**DOI:** 10.3389/fmicb.2024.1458803

**Published:** 2024-07-19

**Authors:** Sujata Sinha, Guneet Kaur, Monika Prakash Rai

**Affiliations:** ^1^Department of Health Research, Indian Council of Medical Research, New Delhi, India; ^2^School of Engineering, University of Guelph, Guelph, ON, Canada; ^3^Department of Biotechnology, Motilal Nehru National Institute of Technology Allahabad, Prayagraj, India

**Keywords:** bioactives, novel biocatalysts, novel microbes, engineered enzymes, bioprocessing, biosensing methods

This Research Topic aimed at getting the latest insight into the production of biomolecules using microbial sources and the development of novel and sensitive microbial detection strategies. Various screening methods for isolation of high-performance strains, yield/productivity enhancement of microbial metabolites such as bioprocess optimization by statistical methods, genetic engineering, and advanced purification methods have been reported. New techniques for identifying improved strains have also been developed and reported to support this. Some of the remarkable microbial research related to the industrial production of metabolites, health, and medicine was published in this Research Topic and is summarized in this editorial.

The production of microbial derived molecules such as exosomes and L-asparaginase and their applications in biomedicine and health such as immunomodulation, gut health, drug delivery, and cancer treatment was reviewed (Tsegaye et al.; Liu). The reviews emphasized on the design of efficient isolation and purification strategies as well as engineering methods for enhanced functionalities of these molecules for biomedical applications. Development and use of *in-silico* methods for rapid screening of effective variants, techniques to assess *in-vivo* distribution and long-term safety was also highlighted considering their applications for human health.

*Bacillus licheniformis* ALSZ2 has been reported (Amin et al.) for lactase production having commercial potential for having lactose intolerant people. Statistical design of media optimization was performed to choose out of 100 strains in the soil polluted with dairy products for isolation of most potent microbial lactase producer with unique lactase capacities. Two level Plackett-Burman design was used for weighing fifteen variables using three factors, lactase activity was enhanced 4 times and 13 U/ml enzyme was achieved.

Genetic engineering strategies for enhanced and tailored production of prodniginines and rhamnolipids has been reported (Kossmann et al.) as biocontrol agents effective against plant nematodes. Bacterial secondary metabolites from genetically engineered *Pseudomonas putida* are of particular interest due to good plant compatibility and low toxicity. These are not as readily accessible so a new hybrid synthetic route was established in the same strain for enhancing the production of bipyrrole precursor and mutasynthesis optimization. Chemically synthesized and supplemented monopyrroles conversion to tripyrrolic compounds and subsequent semisynthesis provided the hydroxylated prodiginine which showed reduced infectiousness in *Arabidopsis thaliana* plants from *Heterodera schachtii*. 50% nematode control was achieved with combined application with rhamnolipids due to the impaired motility and style thrusting of parasites.

Modified yeasts producing proginsenediol-dammarane type ginsenosides, such as G-Rh2, G-Rg3, and G-Rd have been shown to enhance production in *Saccharomyces cerevisiae* (Lin et al.). It was developed by previously reported PnUGT33 as the necessary enzyme for the synthesis and manufacturing of G-Rg3. Two gene modules, LKG and EGH, were built for high level production of desired metabolites. In LKG31 (254.07 mg/L ± 56.49 mg/L) in the optimized YPD (yeast extract, peptone, and dextrose) medium with yeasts overexpressing the ScPGM1, ScPGM2, and ScUGP1 genes, 51 mg/L G-Rg3 production increased threefold. However, this work was unable to be successfully incorporated into the the EGH module (EGH-31 and EGH-53) and efforts to raise the strain titer (56.68 mg/L) after 96 h failed.

Lactic acid bacteria (LAB) (Vasundaradevi et al.) was reported as biocontrol agent to be effective against phytopathogenic fungus. Seven isolates were shown to inhibit its growth, out of which only two obtained from fruits *Ficus* and *Tinospora* exhibited promising antifungal properties against *Alternaria alternata*. Molecular identification technique was used for identifying strains showing higher adaptability to a wide temperature range and salt tolerance of up to 7%. Cell free supernatant (CFS) inhibition was quite high as even 5% crude CFS reduced the fungal growth by >70% while a complete inhibition was reported at 10%. Inhibition was against mycelial growth and conidial germination and it was effective even after long periods of cold storage of CFS.

Human health promoting bioactive compounds were reported from various parts of the medicinal mushroom *Ganoderma lucidum* (Tajik et al.) which include peptides, fatty acids, polysaccharides and triterpenoids. Extracts of isolated compounds from *G lucidum* has been found to be have carcinostatic effects on cancer cell lines like lung, pancreas, breast, skin etc., and triterpenoids have been reported to be the most important bioactive compounds with such effects. Glucose as carbon and corn steep liquor (CSL) as nitrogen source was found to be the best for stimulating the production of a triterpenoid called ganoderic acid based on optimization studies. This was secreted from stem and shell of mushroom and showed substantial anti-microbial and anti-cancer activity in *in-vitro* conditions.

Industrial uses of microbes and ultra-sensitive detection has been revolutionized in the recent times. This is of great importance in the case of anti-microbial resistance. *Pseudomonas aeruginosa*, a principal pathogen responsible for urinary tract infections has been known as difficult to eradicate and has evolved as the main candidate for drug resistance due to the indiscriminate uses of antibiotics. Rapid and efficient detection of microbes as well as their drug resistance genes is desirable for early clinical diagnosis and proper treatment. Recombinant polymerase amplification clustered regularly interspaced short palindromic repeats associated with protein 13a to establish one tube and two step reaction systems for detecting the mexX drug resistance gene in *Pseudomonas* was developed (Zhu et al.). This method outperformed the traditional PCR and qRT-PCR (quantitative Real-time polymerase chain reaction) and limit of detection was as low as 10 aM and 1 aM respectively Primer was specifically designed for drug resistance mexX gene. Method was verified with industrial samples and commercial lateral flow dipstick with LoD of 10 Fm and showed great accuracy.

Design of robust microbial systems as a source of metabolites having commercial value and/or catering to low volume high value products using novel strategies has attracted attention under the sustainable circular economy concept. Ultrasensitive microbial detection in clinical/environmental samples in the areas of health and medicine are of special importance. This Research Topic intends to motivate upcoming researchers to work in these areas which will lead to new innovations and applications in the areas of health, medicine, and industrial (bio)production.

## Author contributions

SS: Conceptualization, Formal analysis, Project administration, Resources, Writing–original draft, Writing–review & editing. GK: Conceptualization, Formal analysis, Writing–original draft, Writing–review & editing, Visualization, Resources. MP: Formal analysis, Conceptualization, Project administration, Writing–original draft, Resources.

